# Intrafamilial Phenotypic Variability in PRPH2-Related Retinopathy

**DOI:** 10.7759/cureus.72580

**Published:** 2024-10-28

**Authors:** Abdelrahman M Elhusseiny, Sairi Zhang, Anna B Sharabura, John R Dehnel, Sami H Uwaydat

**Affiliations:** 1 Ophthalmology, University of Arkansas for Medical Sciences, Little Rock, USA

**Keywords:** choroidal neovascular membrane, peripherin, prph2, rds, retinal dystrophy

## Abstract

Purpose

This study aimed to describe the intrafamilial phenotypic variability and natural history of a *PRPH2*-related retinal dystrophy.

Methods

We performed a retrospective chart review of seven patients from the same family, five of whom had the c.828+3A>T *PRPH2* pathogenic variant, characterizing the natural history and intrafamilial phenotypic variation.

Results

Over the course of nine years, two patients had a deterioration of vision, one had unchanged visual acuity, and four were lost to follow-up after diagnosis. Two patients developed choroidal neovascular membranes. Five family members completed genetic testing.

Conclusions

In the current case series, we described the various phenotypes associated with the *PRPH2* pathogenic variants in related individuals of the same family. We tracked the changes in visual acuity and phenotype in three related patients over five to nine years.

Translational relevance

Studying the natural history and phenotypic variations of the *PRPH2* gene can lead to targeted therapeutic interventions and personalized treatment strategies for affected individuals.

## Introduction

The *PRPH2* (*RDS/peripherin*, OMIM:*179605) gene product is an integral transmembrane glycoprotein that structurally helps form and stabilize photoreceptor discs in rods and cones [1]. The *PRPH2* pathogenic variants are largely autosomal dominant. They can cause a broad spectrum of hereditary retinal dystrophies [1]. Up to 252 variants in the *PRPH2* gene have been identified, an extensive number of which have been confirmed to be pathogenic. The most commonly reported variant types are missense variants affecting >50% of the cases (137 variants), followed by protein-truncating (85 variants), splice site (10 variants), in-frame amino acid insertion/deletion (15 variants), synonymous (three variants), and 5'-3'-untranslated region (two variants) [1-8].

One study has included up to 62 individuals with the c.828+3A>T *PRPH2* pathogenic variant, a common splice site mutation [9]. This specific pathogenic variant has complete penetrance within families and results in multiple different phenotypes within families [9]. Shankar et al. identified four distinct phenotypes consistent with pattern dystrophies, cone-rod dystrophies, retinitis pigmentosa, or central areolar chorioretinal dystrophy with extensive intra- and interfamilial variation, determined a founder effect with this pathogenic variant, and characterized potential modifiers that explain intrafamilial variation [10]. The founder effect of the *PRPH2* splice site mutation c.828+3A>T has been well established. The aberrant *PRPH2* transcript results in an abnormal truncated protein product that cannot be transported to the outer segments of the photoreceptors, leading to loss of photoreceptor function and eventual cell death. However, the authors did not elaborate on the long-term follow-up. Therefore, the natural history of the c.828+3A>T *PRPH2* pathogenic variant is still unclear [9-11]. We herein present this series of seven family members, five of whom had the c.828+3A>T *PRPH2* pathogenic variant, with the goal of understanding the natural history and the intrafamilial phenotypic variation across seven individuals in the same family.

## Materials and methods

Subjects and clinical studies

The study was approved by the Institutional Review Board of the University of Arkansas for Medical Sciences (approval number: 228531) and was conducted in accordance with the tenets of the Declaration of Helsinki.

We conducted a retrospective chart review of all patients from the same family who resided in Arkansas and agreed to come to the Jones Eye Institute at the University of Arkansas for Medical Sciences. The index patient was first identified in 2013 with the c.828+3A>T *PRPH2* pathogenic variant using the Casey Eye Institute (Portland, Oregon) Stargardt/macular dystrophy panel. Direct testing for the c.828+3A>T mutation was done on the other family.

Data collected included age at the time of presentation, gender, best-corrected visual acuity (BCVA) at the initial visit and last follow-up, slit-lamp findings, fundus examination findings, electrophysiological testing results (when available), and genetic testing results. The BCVA was recorded using standard Snellen visual acuity charts.

Electrophysiology

Patients were dilated using tropicamide 1% and phenylephrine 2.5% and then dark-adapted for 45 minutes before testing. The electroretinogram (ERG) was recorded from the dilated, dark-adapted eye following the International Society for Clinical Electrophysiology of Vision (ISCEV) standards, using a UTAS SunBurst system (LKC Technologies, Gaithersburg, Maryland, United States).

Statistical analysis

We entered data and analyzed it using Microsoft Excel 2019 (Microsoft Corporation, Redmond, Washington, United States). Quantitative data were expressed as median, mean±standard deviation, and range.

## Results

**Figure 1:**
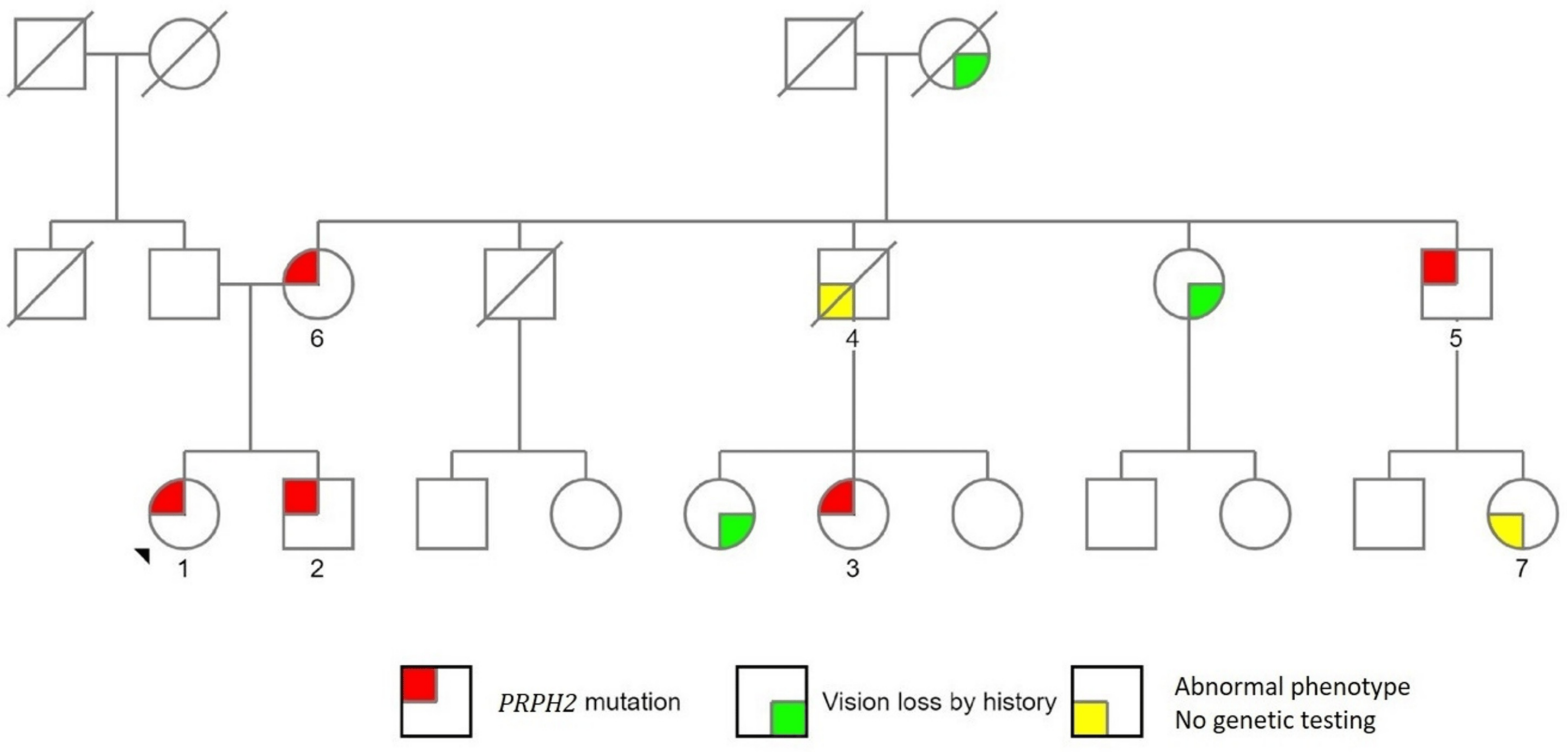
PRPH2 family pedigree. The family tree for the index patient (black arrow) and patients noted in this study. Circles are women, and squares represent men. A diagonal line signifies a deceased individual. The red color fill represents confirmed PRPH2 mutation by genetic testing. The green color fill represents a history of vision loss due to progressive retinal atrophy attributed to the PRPH2 mutation. Patient identifiers are present as necessary. Five family members have confirmed genetic testing, and five members have significant vision loss per history. The mutation appears to be passed in an autosomal dominant fashion, beginning with the index patient's grandmother, who is not confirmed to have the genetic mutation but has significant vision loss.

The current study included seven patients with a mean age of 62.8±11 years (median: 58 years) at the time of presentation to our clinic (Table 1). Four patients (57.1%) were women. Of seven patients, three were lost to follow-up, and one subsequently passed away. The remaining three patients (patients #1, #3, and #7) were followed up for nine, six, and five years, respectively. Initial BCVA ranged from 20/25 (57%) to hand motion. At the final follow-up visit, BCVA deteriorated in patients #1 and #7; however, it was stable in patient #3. Patients were referred to our clinic with a variety of diagnoses, including retinal dystrophy (two patients), rod-cone dystrophy (one patient), macular degeneration (three patients), and rod dysfunction (one patient). Five patients completed genetic testing. The index patient (patient #1) and her mother (patient #6) developed choroidal neovascular membranes (CNVMs). Table 2 summarizes the baseline characteristics and the natural course of the disease in the whole cohort. The family pedigree detailing the presence of the *PRPH2* pathogenic variant and the status of vision loss is available in Figure 1.

**Table 1:** Statistical measures of the quantitative data. *: For logMAR VA at the last follow-up, three patients were lost to follow-up and therefore not included in the statistics.

	Age (years)	Initial logMAR VA	Last logMAR VA*
Mean (SD)	62.86 (11.01)	0.7 (0.89)	0.39 (0.37)
Median	58	0.1	0.2
Range	30	2.3	0.9

**Table 2:** Summary of the baseline characteristics and natural course of the whole cohort. BCVA: best-corrected visual acuity; OD: right eye; OS: left eye; OU: both eyes; CNVM: choroidal neovascular membrane; CF: counting fingers; HM: hand motion *: The patient developed a choroidal neovascular membrane one year after her initial presentation to our clinic. She was treated with two intravitreal bevacizumab injections with no recurrence.

Patient case number	Age (years)	Gender	Relation to index patient	Initial BCVA	Length of follow-up	Genetic testing (variant zygosity)	Progression of the disease	BCVA at the last follow-up
1	49	Female	Self	OU: 20/25	9 years	c.828+3A>T	CNVM OS*	OU: 20/200
2	53	Male	Brother	OU: 20/25	1 year	c.828+3A>T	Stable VA	OU: 20/25
3	58	Female	Maternal cousin	OU: 20/25	6 years	c.828+3A>T	Stable VA	OU: 20/25
4	79	Male	Maternal uncle	OD: 20/200; OS: CF 10 ft	None	Not done	Not available	Lost to follow-up
5	75	Male	Maternal uncle	OU: HM	None	c.828+3A>T	Not available	Lost to follow-up
6	71	Female	Mother	OD: 20/25; OS: CF 3 ft	None	c.828+3A>T	Not available	Lost to follow-up
7	55	Female	Maternal cousin	OU: 20/25	5 years	Not done	Decline in VA	OD: 20/50; OS: 20/40

Patient #1 (index patient)

**Figure 2:**
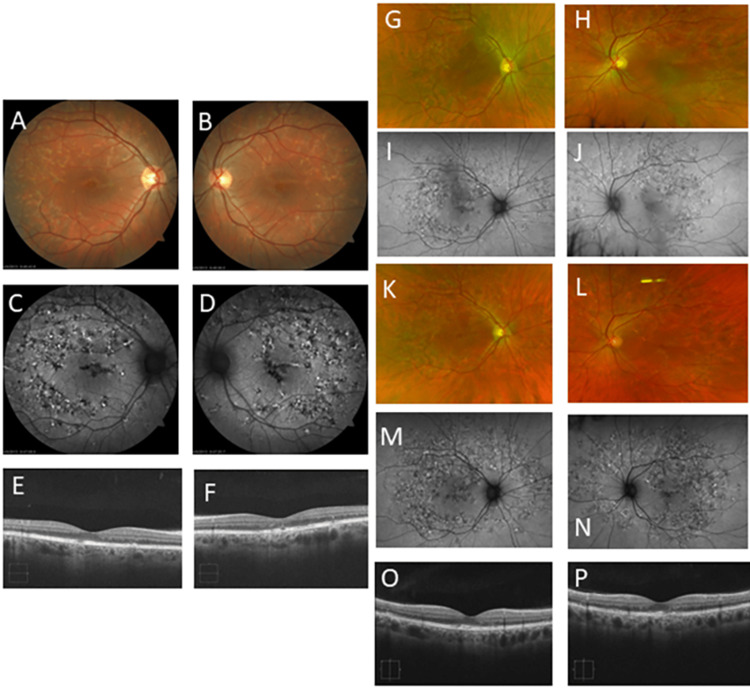
Patient #1 imaging. Fundus photographs and FAF of both eyes of patient #1 in 2013 showing macular pisciform lesions (A-D). OCT of both eyes in 2013 shows RPE thickening (E-F). Fundus photographs and FAF of both eyes reveal that the number and distribution of the yellow pisciform deposits increased over three years from 2016 (G-J) to 2019 (K-N). Serial OCT exhibited progressive RPE clumping/deposits or thickening and gradual loss of outer retinal layers in both eyes in 2019 (O-P). FAF: fundus autofluorescence; OCT: optical coherence tomography; RPE: retinal pigment epithelium

A 49-year-old Caucasian woman presented to our clinic in 2013 complaining of increasing blind spots in both eyes over 20 years. She was diagnosed with presumed ocular histoplasmosis by one provider and Stargardt disease by another. Her family history was significant for glaucoma in her father, and multiple family members were diagnosed with macular degeneration. At the initial presentation, her BCVA was 20/25 in each eye. Her fundus examination revealed diffuse pisciform lesions in the posterior pole with speckled autofluorescence (Figure 2A-2D). Spectral-domain optical coherence tomography (OCT) using the Cirrus 5000 system (Carl Zeiss Meditec, Dublin, California, United States) demonstrated uniform retinal pigment epithelium (RPE) thickening in both eyes, with localized areas of RPE loss (Figure 2E, 2F). Full-field electroretinography (ffERG) demonstrated reduced rod amplitudes with normal implicit times bilaterally and normal cone responses. Genetic testing revealed a c.828+3A>T pathogenic variant in the *PRPH2* gene.

Since receiving this diagnosis in 2013, her BCVA declined from 20/25 to 20/200 in each eye over nine years. The patient's disease course was complicated in 2014 by developing a sub-macular CNVM in the left eye, confirmed by fluorescein angiography (FA). She received two intravitreal injections of bevacizumab spaced over two months with complete resolution of the CNVM and no recurrence to date. In 2016, the patient elected to undergo intravitreal injections of bone marrow-derived stem cells in both eyes in another facility; however, she experienced no visual improvement following this treatment. Serial OCT exhibited progressive focal RPE atrophy, with gradual loss of the overlying outer retinal layers in both eyes. Throughout her follow-up, she developed new yellow pisciform lesions extending from the posterior pole to the periphery (Figure 2G-2P).

Patient #2

**Figure 3:**
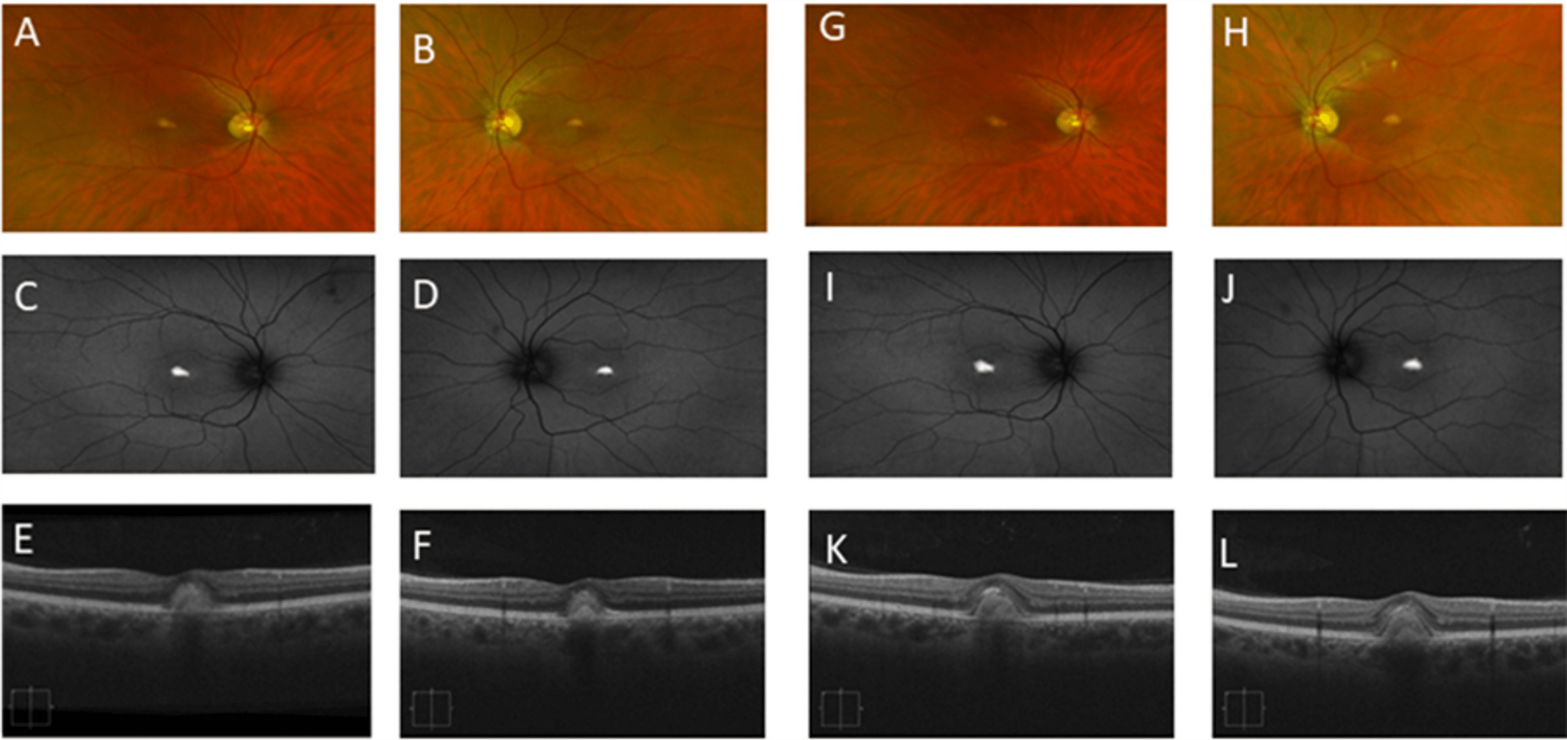
Patient #2 imaging. Fundus examination (A, B, G, H) revealed yellow retinal lesions in the macula of both eyes that localized to the sub-retinal space. FAF of the right eye (C and I) and left eye (D and J) revealed an area of macular hyper-autofluorescence. OCT (E, F, K, L) shows a sub-macular hyperreflective area corresponding to yellow deposits seen on fundus examination, increasing in size over one year from 2019 (A-F) to 2020 (G-L). FAF: fundus autofluorescence; OCT: optical coherence tomography

A 53-year-old Caucasian man, brother to the index patient, presented with visual acuity of 20/25 in each eye. Fundus examination (Figure 3A, 3B, 3G, 3H) revealed yellow retinal lesions in the macula of both eyes that localized to the sub-retinal space. Fundus autofluorescence (FAF) of the right eye (Figure 3C, 3I) and left eye (Figure 3D, 3J) revealed an area of macular hyper-autofluorescence. OCT (Figure 3E, 3F, 3K, 3L) shows a sub-macular hyperreflective area corresponding to yellow deposits seen on fundus examination.

Patient #3

**Figure 4:**
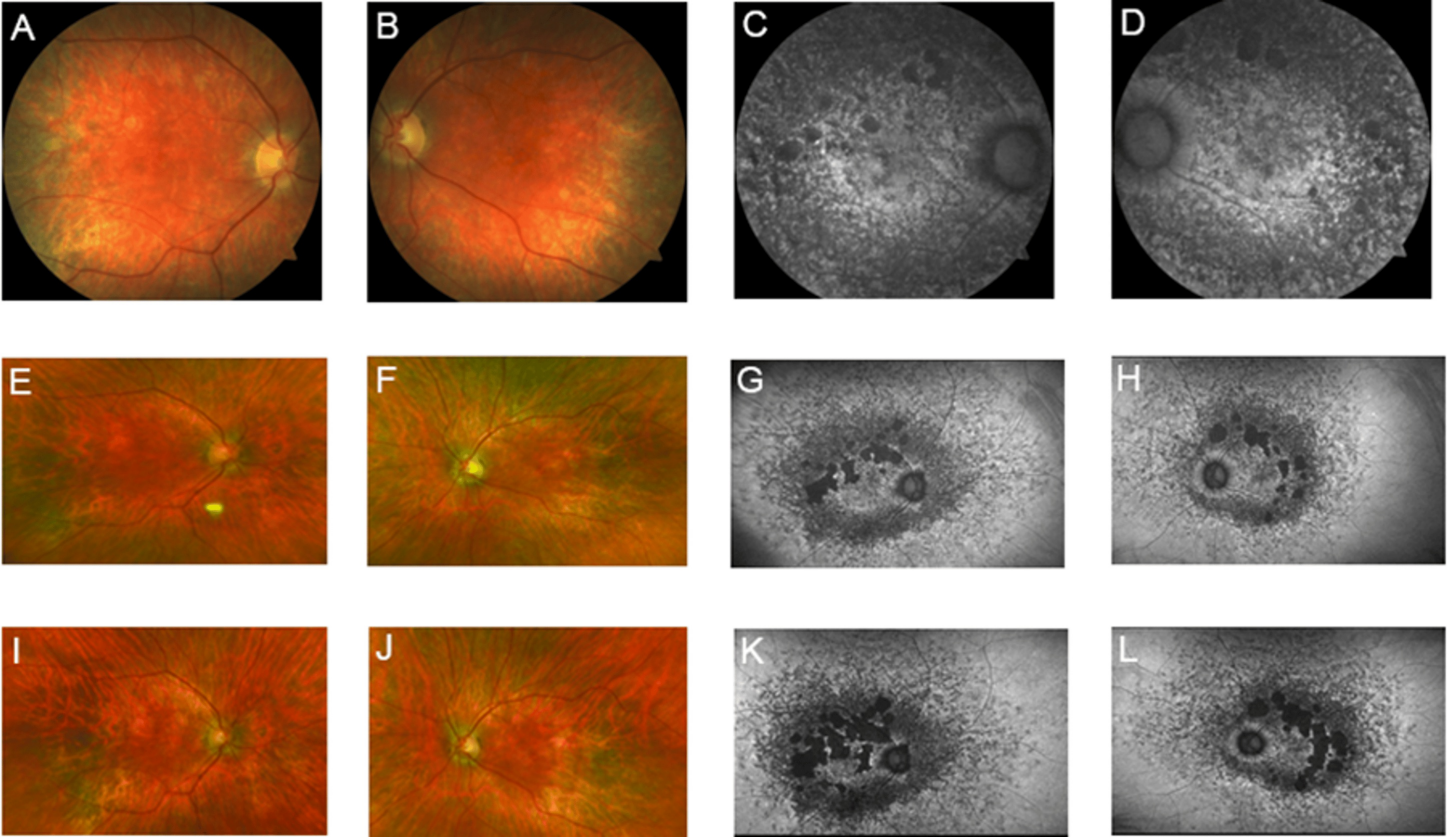
Patient #3 imaging. Wide-field fundus imaging and FAF for patient #3 over six years: 2013 (A-D), 2016 (E-H), and 2019 (I-L). Fundus pictures of both eyes (A, B, E, F, I, J) show round areas of macular atrophy, pigment changes, and mid-peripheral RPE atrophy in both eyes progressing over three years. FAF of both eyes (C, D, G, H, K, L) showing patches of hypo-autofluorescence in the mid-periphery of the retina bilaterally progressing over three years. FAF: fundus autofluorescence; OCT: optical coherence tomography; RPE: retinal pigment epithelium

A 58-year-old Caucasian woman, the cousin to patient #1, presented with a BCVA of 20/25 in each eye. Fundus examination revealed patchy areas of RPE atrophy in the posterior pole and along the arcades, with yellow retinal deposits in the periphery in both eyes (Figure 4A, 4B, 4E, 4F, 4I, 4J). FAF highlighted the patches of hypo-autofluorescence in the mid-periphery of the retina bilaterally (Figure 4C, 4D, 4G, 4H, 4K, 4L). Spectral-domain OCT revealed atrophy of the outer retinal layers in both eyes in the area of atrophy, with a thickened RPE layer in the unaffected areas. ffERG testing showed that rods had reduced amplitudes and delayed implicit times, while cones had normal amplitudes but delayed implicit times, consistent with a rod-cone dysfunction. Genetic testing revealed a c.828+3A>T pathogenic variant in the *PRPH2* gene.

The patient was followed for six years following the initial presentation, during which she had a stable BCVA. She developed an inferior schisis of her left retina. Serial imaging showed progressive atrophy of both eyes' RPE and outer retinal layers (Figure 4). Repeat ffERGs showed worsening retinal function with continued deterioration of rod and, eventually, cone responses.

Patient #4

A 79-year-old man, the maternal uncle of patient #1, presented with a BCVA of 20/200 in the right eye and counting fingers at 10 feet in the left eye. Fundus examination disclosed extensive chorioretinal atrophy in the posterior segment of both eyes. He did not receive genetic testing and was lost to follow-up.

Patient #5

**Figure 5:**
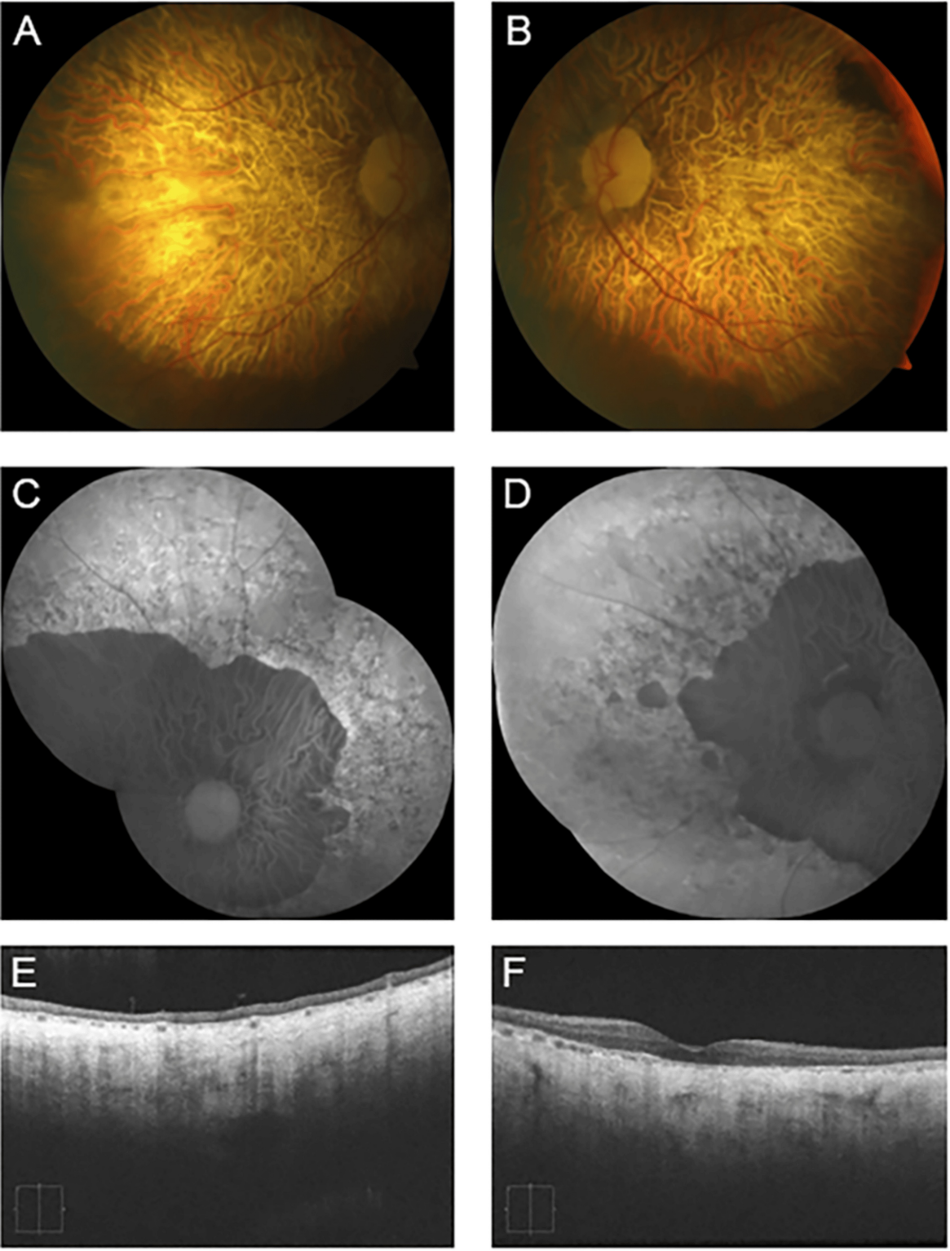
Patient #5 imaging. Fundus examination (A and B) was significant for macular atrophy in both eyes and areas of atrophy on the peripheral retina bilaterally. FAF (C and D) showed a large area of hypo-autofluorescence in both eyes involving the macula. OCT (E and F) revealed retinal atrophy, more evident in the right eye. FAF: fundus autofluorescence; OCT: optical coherence tomography

A 75-year-old Caucasian man, the cousin to the index patient, presented with a BCVA of hand motion in each eye. Fundus examination (Figure 5A, 5B) was significant for macular atrophy in both eyes and areas of atrophy on the peripheral retina bilaterally.

Patient #6

A 71-year-old female patient, the mother of the index patient, was referred for the evaluation of bilateral macular degeneration. Her presenting BCVA was 20/25 in the right eye and counting fingers at 3 feet in the left eye. Fundus examination disclosed pigmented pisciform lesions in the periphery of both eyes and central foveal atrophy in the left eye.

Patient #7

**Figure 6:**
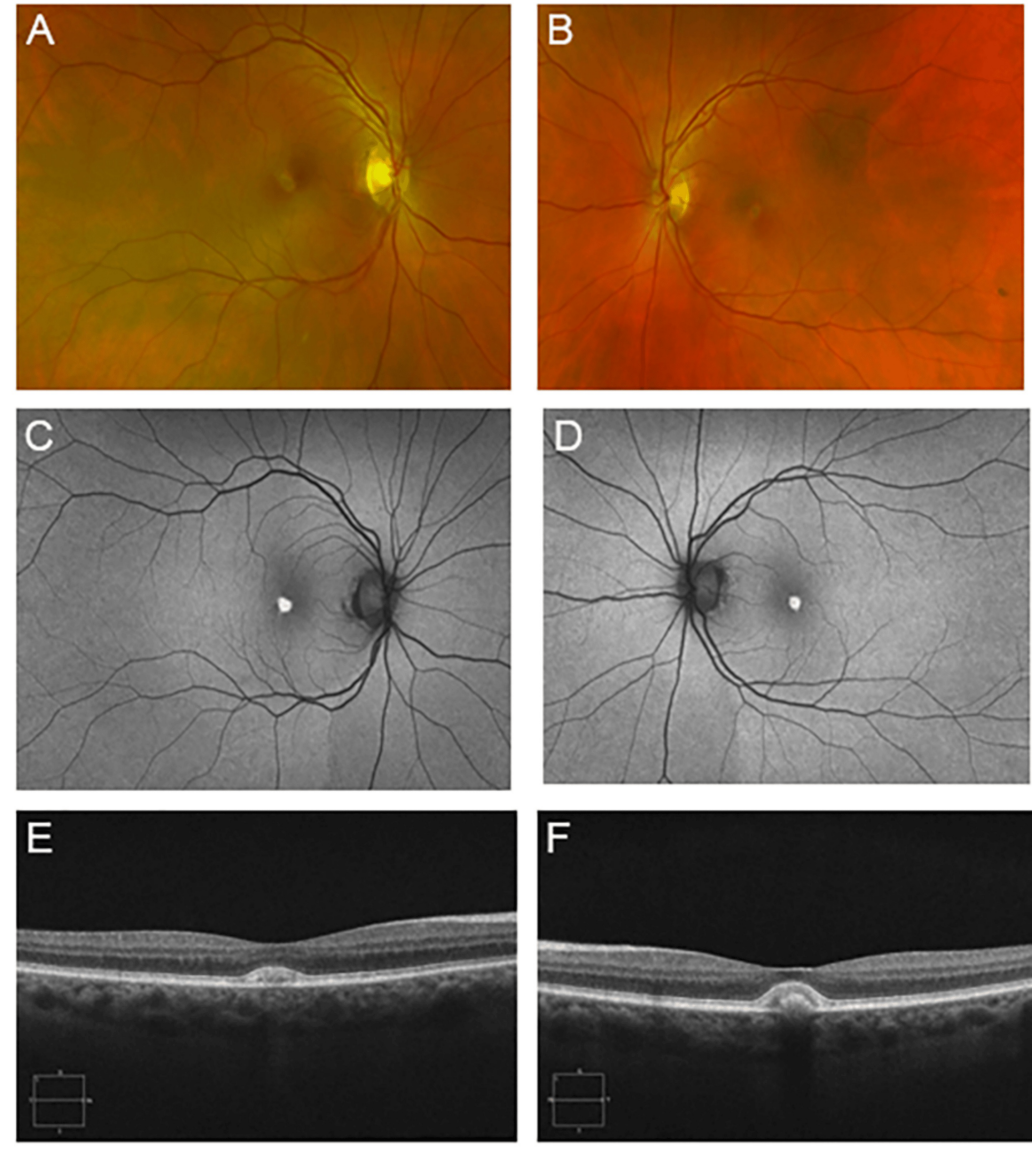
Patient #7 imaging. Fundus photographs, FAF, and OCT of the macula of the right eye (A, C, E) and left eye (B, D, F) of patient #7. Fundus examination (A and B) revealed large deposits under the fovea with pigment clumping in the right eye, while her left retina exhibited RPE changes with yellow deposits. FAF (C and D) revealed sub-foveal hyper-autofluorescence. OCT (E and F) shows sub-foveal RPE thickening and deposits, with the left eye larger than the right eye. FAF: fundus autofluorescence; OCT: optical coherence tomography; RPE: retinal pigment epithelium

A 55-year-old Caucasian woman, cousin to the index patient, with an ocular history of macular degeneration diagnosed at age 40, presented to the clinic due to episodic blurry vision, wavy spots, and occasional black spots in both eyes. BCVA at the time of her initial visit was 20/25 in each eye. Fundus examination was significant for pattern dystrophy bilaterally. Over the next five years, her visual acuity worsened slightly to 20/50 in the right eye and 20/40 in the left. Fundus examination (Figure 6A, 6B) revealed large deposits under the fovea with pigment clumping in the right eye, while her left retina exhibited RPE changes with yellow deposits. FAF (Figure 6C, 6D) revealed foveal hyper-autofluorescence, indicating excessive accumulation of lipofuscin.

## Discussion

We present a case series of seven patients of the same family, five of whom had the c.828+3A>T *PRPH2* splice site pathogenic variant. This specific pathogenic variant has complete penetrance within families and results in multiple different phenotypes. In addition, we had a long-term follow-up for three of our patients for five to nine years with FAF, OCT, and fundus photographs. This enabled us to detect the progressive changes, giving insights into the natural history of the disease. Diagnosis of *PRPH2*-associated dystrophy without genetic testing, ffERG, and FAF is challenging mainly because of the phenotypic variations and changing characteristics of the lesion throughout the disease course. This was evident in our cohort, in which three patients were referred to us for age-related macular degeneration and one patient was diagnosed with Stargardt disease. Boon and colleagues demonstrated that different *PRPH2* pathogenic variants could be present with multifocal pattern dystrophy simulating Stargardt disease [4]. Renner and colleagues described the phenotypic variability in 22 patients with the *PRPH2* pathogenic variant. Out of 22 patients, nine were diagnosed with central areolar choroidal dystrophy, seven were diagnosed with autosomal dominant retinitis pigmentosa, three were diagnosed with adult vitelliform macular dystrophy, and three were diagnosed with cone-rod dystrophy [8]. Due to the phenotypic variability of *PRPH2*-associated dystrophy, diagnosis is challenging. Though there is no treatment currently available, correctly diagnosing the condition will better inform future targeted treatment options and decisions on inclusion in ongoing clinical trials.

In our study, five patients had the c.828+3A>T *PRPH2* splice site pathogenic variant. Shankar and colleagues studied the *PRPH2* pathogenic variant in 97 individuals and concluded that the c.828+3A>T *PRPH2* splice site pathogenic variant has a founder effect and is a frequent cause of retinal dystrophies [9]. However, the authors did not elaborate on the long-term follow-up [10]. Keen and Inglehearn demonstrated that missense pathogenic variants and small in-frame deletions in the *PRPH2* gene in the large intradiscal loop between the third and fourth transmembrane proteins usually result in severe phenotypes such as autosomal dominant retinitis pigmentosa or severe macular dystrophy [12]. However, nonsense pathogenic variants were associated with milder phenotypes.

Phenotypic variability in *PRPH2*-related retinal degeneration is attributed to the presence of other genetic modifiers. Potential modifiers include pathogenic variants of the *ROM1* and *ABCA4* genes, but other mutant alleles and unidentified modifiers can affect the phenotypic expression of the *PRPH2* pathogenic variant [13]. In our series, information on potential modifier genes was not available.

In our cohort, two patients had CNVMs. Few studies have reported the association of CNVMs in patients with the *PRPH2* pathogenic variants. Moshfeghi and colleagues reported sub-foveal CNVM in three patients with adult-onset foveomacular dystrophy caused by *RDS/PRPH2* gene pathogenic variants [14].

Patient #1 had an intravitreal injection of bone marrow-derived stem cells bilaterally at an outside clinic. The patient did not have any significant visual improvement or develop any adverse events. To date, there is no scientifically proven evidence that stem cell injections can alter the course of inherited or non-inherited retinal degeneration. However, there have been studies to determine the safety of such injections. In a phase 1 trial, Siqueira and colleagues injected autologous bone marrow-derived mononuclear cells intravitreally in five patients (three patients with advanced retinitis pigmentosa and two patients with cone-rod dystrophy) [15]. The authors reported improved BCVA in four out of five patients by one line. The BCVA improvement occurred one week after injection and continued for 10 months. No functional or structural adverse events were reported over the 10-month follow-up period [15]. Another series by Satarian and colleagues evaluated the safety of intravitreal injection of bone marrow-derived mesenchymal cells in three eyes with advanced retinitis pigmentosa [16]. Two eyes reported improved light perception over three months after injection with no adverse events. However, the third eye developed subsequent tractional retinal detachment [16].

The current study is limited by the small sample size and its retrospective nature. Another limitation is the inability to perform whole exome sequencing to detect potential modifying factors. However, due to financial constraints, only targeted genetic testing was completed. Imaging modalities were not consistent across all patients, given that the imaging machines were upgraded over the observation period.

## Conclusions

Overall, this retrospective case series strengthens the understanding of the long-term retinal disease progression in patients with a common *PRPH2* pathogenic variant. By tracking the variation in BCVA and phenotype in patients over the years, this study illustrates the variable expressivity of disease in a single affected family. Out of three patients with long-term follow-up, BCVA slowly deteriorated in two patients and was preserved in one patient. Further analysis of similar families would aid in describing the natural history of the *PRPH2* pathogenic variants over time.

## References

[1] Boon CJ, den Hollander AI, Hoyng CB, Cremers FP, Klevering BJ, Keunen JE (2008). The spectrum of retinal dystrophies caused by mutations in the peripherin/RDS gene. Prog Retin Eye Res.

[2] Ahmad OR, Ayyagari R, Zacks DN (2010). A novel missense mutation in the rds/peripherin gene associated with retinal pattern dystrophy. Retin Cases Brief Rep.

[3] Kalyanasundaram TS, Black GC, O'Sullivan J, Bishop PN (2009). A novel peripherin/RDS mutation resulting in a retinal dystrophy with phenotypic variation. Eye (Lond).

[4] Boon CJ, van Schooneveld MJ, den Hollander AI (2007). Mutations in the peripherin/RDS gene are an important cause of multifocal pattern dystrophy simulating STGD1/fundus flavimaculatus. Br J Ophthalmol.

[5] Passerini I, Sodi A, Giambene B, Menchini U, Torricelli F (2007). Phenotypic intrafamilial variability associated with S212G mutation in the RDS/peripherin gene. Eur J Ophthalmol.

[6] Duncan JL, Talcott KE, Ratnam K (2011). Cone structure in retinal degeneration associated with mutations in the peripherin/RDS gene. Invest Ophthalmol Vis Sci.

[7] Conley SM, Stuck MW, Watson JN, Naash MI (2017). Rom1 converts Y141C-Prph2-associated pattern dystrophy to retinitis pigmentosa. Hum Mol Genet.

[8] Renner AB, Fiebig BS, Weber BH (2009). Phenotypic variability and long-term follow-up of patients with known and novel PRPH2/RDS gene mutations. Am J Ophthalmol.

[9] Shankar SP, Hughbanks-Wheaton DK, Birch DG (2016). Autosomal dominant retinal dystrophies caused by a founder splice site mutation, c.828+3A>T, in PRPH2 and protein haplotypes in trans as modifiers. Invest Ophthalmol Vis Sci.

[10] Shankar SP, Birch DG, Ruiz RS (2015). Founder effect of a c.828+3A>T splice site mutation in peripherin 2 (PRPH2) causing autosomal dominant retinal dystrophies. JAMA Ophthalmol.

[11] Lee CS, Leys M (2020). A family affected by novel C213W mutation in PRPH2: long-term follow-up of CNV secondary to pattern dystrophy. Ophthalmic Surg Lasers Imaging Retina.

[12] Keen TJ, Inglehearn CF (1996). Mutations and polymorphisms in the human peripherin-RDS gene and their involvement in inherited retinal degeneration. Hum Mutat.

[13] Conley SM, Naash MI (2014). Gene therapy for PRPH2-associated ocular disease: challenges and prospects. Cold Spring Harb Perspect Med.

[14] Moshfeghi DM, Yang Z, Faulkner ND (2006). Choroidal neovascularization in patients with adult-onset foveomacular dystrophy caused by mutations in the RDS/peripherin gene. Adv Exp Med Biol.

[15] Siqueira RC, Messias A, Voltarelli JC, Scott IU, Jorge R (2011). Intravitreal injection of autologous bone marrow-derived mononuclear cells for hereditary retinal dystrophy: a phase I trial. Retina.

[16] Satarian L, Nourinia R, Safi S (2017). Intravitreal injection of bone marrow mesenchymal stem cells in patients with advanced retinitis pigmentosa; a safety study. J Ophthalmic Vis Res.

